# Emergency nurses’ ways of coping influence their ability to empower women to move beyond the oppression of intimate partner violence

**DOI:** 10.4102/phcfm.v8i2.957

**Published:** 2016-04-15

**Authors:** Annatjie van der Wath, Neltjie van Wyk, Elsie Janse van Rensburg

**Affiliations:** 1 Department of Nursing Science, University of Pretoria, South Africa; 2 Department of Health Studies, University of South Africa, South Africa

## Abstract

**Background:**

Millennium Developmental Goal 3 (MDG 3) aims at enhancing gender equity and empowerment of women. Emergency nurses who often encounter women injured by their intimate partners are at risk of developing vicarious traumatisation, which may influence their ability to empower women to move beyond the oppression of intimate partner violence.

**Aim:**

This article aims to, (1) describe emergency nurses’ ways of coping with the exposure to survivors of intimate partner violence, and (2) recommend a way towards effective coping that will enhance emergency nurses’ abilities to empower women to move beyond the oppression of intimate partner violence to contribute to the achievement of MDG 3.

**Setting:**

The study was conducted in emergency units of two public hospitals in an urban setting in South Africa.

**Method:**

A qualitative design and descriptive phenomenological method was used. Emergency nurses working in the setting were purposively sampled and interviewed. The data were analysed by searching for the essence and meaning attached to the exposure of emergency nurses to survivors of intimate partner violence.

**Results:**

Emergency nurses’ coping responses were either aimed at avoiding or dealing with their exposure to survivors of intimate partner violence. Coping aimed at dealing with the exposure included seeking support, emotion regulation and accommodative coping.

**Conclusion:**

Emergency nurses employ either effective or ineffective ways of coping. Less effective ways of coping may increase their risk of vicarious and secondary traumatisation, which in turn may influence their ability to empower women to move beyond the oppression of intimate partner violence.

## Introduction

Emergency nurses are often in contact with survivors of intimate partner violence (IPV).^1,2^ In this article, the term ‘survivor of IPV’ refers to a female health care user seeking help at an emergency department for health problems related to exposure to physical, psychological and sexual violence inflicted by a current or former male intimate partner.^3,4^ By using the term ‘survivor’, the authors acknowledge a woman’s potential to move beyond the oppression of IPV when empowered with the necessary knowledge and assistance.

Towards achieving Millennium Development Goal 3 (MDG 3) of promoting gender equality and empowering women, one of the key working areas identified was the response to gender-based violence inflicted by a partner.^5^ Ending violence against women is described as the ‘missing Millennium Development Goal (MDG)’. Policies and programmes to address violence against women are key areas in achieving in MDG 3.^6^ Even if exposed to seriously injured survivors, nurses are expected to suppress their emotional responses, stay non-judgmental and provide empathetic care.^7^ In order to cope with sometimes overwhelming emotional distress, nurses might attempt to avoid situations where they are required to provide care to survivors of IPV.^7^ This may result in hindering the attaining of the MDG 3 as avoidance creates a barrier to empowering women to move beyond the oppression of IPV. Positive and active ways of coping (for example, spirituality and seeking social support) were associated with nurses’ resilience to the effects of exposure to traumatic work situations.^8^

Frequent exposure to survivors of IPV may put nurses at risk of developing secondary traumatic stress.^9^ In order to moderate the impact of serious events on nurses, research needs to explore the way nurses cope with these events.^10^ Studies underscore the importance of understanding stress and coping in the nursing population in order to develop supportive strategies for nurses.^11,12^

A real-life event appraised by an individual as a demand threatening to exceed his or her available resources may result in stress. ‘Coping’ reflects a person’s cognitive and behavioural attempts to alter the problems caused by a stressful event and regulate the stressful, and sometimes intense, emotional responses. Coping with a successful outcome will produce positive emotions whilst a less favourable outcome will lead to negative emotions.^13,14^

A stressful event emergency nurses often need to cope with is the exposure to caring for survivors of IPV. American studies reported a 17%^15^ and 32%^1^ prevalence rate of IPV amongst women seeking help at emergency departments, whilst an Australian study reported a 14% prevalence rate.^16^ Similar South African statistics are not available, but in a country where a woman is killed by her intimate partner every eight hours,^17^ emergency nurses would often be expected to care for survivors of IPV.

Exposure to stressful and traumatic incidents such as IPV may lead to cognitive, behavioural and affective changes in the way emergency nurses relate to others and themselves.^18,19^ Vicarious and secondary traumatisation amongst helping professionals is well described in the literature. Nurses are at risk of developing, secondary traumatisation (also known as ‘compassion fatigue’),^21^ burnout^22^ and post-traumatic stress,^23^ in particular when they are constantly exposed to and aware of patients’ suffering^20^ but feel helpless or lack the resources to intervene.^24^ Vicarious traumatisation, coined by McCann and Pearlmann,^25^ is associated with disruptions in memory and cognitive schemas. Secondary traumatisation, described by Figley,^26,27^ is characterised by symptoms similar to those of post-traumatic stress disorder, the only difference being that trauma is experienced by a significant other. Vicarious and secondary traumatisation affect helping professionals’ well-being and compassion^28^, deplete their ability to bear others’ suffering and feel and care for them.^29^

Health care professionals’ ways of coping play a role in the prevention and development of vicarious and secondary traumatisation.^30,31^ Ways of coping such as self-care strategies, seeking support from others, caregiving skills and conflict resolution can reduce professionals’ chances of developing vicarious traumatisation.^32,33^

Emergency nurses are expected to identify and respond to survivors of IPV in an attempt to address MDG 3.^34,35^ Such exposure might be perceived as a stressful event^7,9^ with potential negative outcomes for the emergency nurses. The question asked in the research on which this article is based was how emergency nurses cope with the stressful event of being exposed to survivors of IPV.

### Purpose of the study

The aim of this article is to describe emergency nurses’ ways of coping with the experience of caring for survivors of IPV and to discuss recommendations based on emergency nurses’ experiences.

## Research methods and design

### Study design

The research followed a qualitative design and a descriptive phenomenological approach. Guided by the phenomenological philosophy that knowing is grounded in the life-world as the inter-subjective context of human experience,^36,37^ the research aimed to illuminate emergency nurses’ attempts to cope with the exposure to survivors of IPV, which may affect their ability to empower these women to move beyond the oppression of IPV.

### Setting

The study was conducted in emergency units of two public hospitals in an urban setting in South Africa. Formal statistics are not kept, but informal discussions revealed that nurses working in emergency units in South Africa are often required to provide care to female survivors of IPV.

### Study population and sampling strategy

The study population was emergency nurses working for at least one year in the emergency units of two public hospitals in an urban area in South Africa. Considering that male and female nurses’ ways of coping with the exposure to IPV perpetrated against women may differ, the researcher decided to select only female participants. Emergency nurses were informed about the study. The researcher purposively selected emergency nurses willing to participate until data saturation occurred and no new themes emerged.^38^ Nine female participants, four from one hospital, and five from another hospital, ages ranging between 26 and 50 years, were selected and interviewed.

### Data collection

Data were collected through unstructured interviews conducted in a private room in the hospital. Broad open-ended questions were asked; for example, *How do you cope when you are required to care for a woman exposed to IPV? How do you feel about caring for women exposed to IPV?* In-depth data were obtained with the use of interviewing skills such as reflection and paraphrasing. Considering the emotional and sensitive nature of the research phenomenon, participants were supported and referred for counselling as needed. Guided by data saturation, nine interviews lasting 45 to 60 minutes were conducted. Descriptive and reflective field notes were used to supplement the interview data and increase the interviewer’s awareness of her personal values and possible areas of bias or role conflict.^38^

### Data analysis

Descriptive phenomenological data analysis was pursued to explore the meaning of participants’ responses.^37^ A general sense of the whole was obtained whilst reading through the transcribed interviews. By using free imaginative variation, the researcher followed the concrete experiences obtained from participants, reflected on, and divided, the data into meaning units according to the different ways of coping used by participants.

## Ethical considerations

Approval to conduct the study was obtained from the Ethics Committee of the Faculty of Health Sciences, University of Pretoria (ref: 125/2010) and the hospital management. In line with ethical research principles, informed consent was obtained and participants were informed of their right to withdraw at any time without any consequences.

### Rigor

Rigor of the findings was ensured through bracketing, reflexivity and eidetic reduction. The researcher, a psychiatric nurse, bracketed her prior knowledge and assumptions regarding the coping models used to assist clients who have been exposed to trauma. She analysed and reflected on her self-awareness during the research process and recorded these as reflective notes.^36^ Eidetic reduction entailed the uncovering of the essential characteristics of participants’ experiences. The findings reflect the meaning of coping with the exposure to survivors of IPV quoted in participants’ words.

### Results

In essence participants’ ways of coping were either aimed at escaping or dealing with their exposure to survivors of IPV. Ways to escape the emotions related to care for a survivor of IPV included avoidance, whilst attempts to deal with the experience included seeking support, regulation of emotions and accommodation. Participants used suppression of emotions in an attempt to regulate their emotions. Being unable to change or control IVP as an inevitable phenomenon in an emergency unit, participants used accommodative ways of coping, namely distraction, spirituality, acceptance and meaning making.

### Coping: Avoidance

A way of coping was to try to avoid exposure to survivors of IPV:

‘I hear the patient explaining the situation that occurred to the sister … it makes me stay away from that unit until that patient leaves.’ (Participant 3, female, 27)

Avoidance also occurred on an emotional level when a participant attempted not to experience any emotions:

‘… in order to work with this person or to help this person I distance myself … I think that’s in order not to experience anger …’ (Participant 7, female, 26).

### Coping: Dealing with the experience of being exposed to survivors of intimate partner violence

Participants’ attempts to deal with their experiences included *seeking support, regulation of emotions* and *accommodation*.

### Seeking support

Participants who used support-seeking verbalised that ‘it feels better’ to talk to colleagues as talking provides a way of ventilating feelings:

‘I can come here and say to Sister X: “You know what Sister X? We have got this patient. This is what happened to this patient; this is so sad.”’ (Participant 1, female, 32)

Emergency nurses also talked to the spiritual counsellors who provide support to patients and hospital staff on a voluntary basis:

‘… he is here for us … you just go there and talk to them … It’s good for us … the spiritual counsellors … and then after that I’m back to normal.’ (Participant 8, female, 46)

### Regulation of emotions

Participants attempted emotion regulation as a way of coping. They sometimes cried when they were emotionally touched by a case:

‘I will go to the bathroom, I cry then I come back and feel a little bit relieved afterwards.’ (Participant 3, female, 27)

Crying was not so much seen as a way of coping but rather as something happening when one is not able to control one’s emotions:

‘… there are experiences that you get to the extent where you cannot hold yourself – you will end up crying …’ (Participant 1, female, 32)

Crying was not something participants wanted others to see: ‘You will just like wipe your tears … and say:

‘… people must not see that I am crying.’; ‘… going somewhere to hide yourself from people not seeing you are hurt’; ‘… but isn’t it you won’t let others see; you just cry internally?’ (Participant 4, female, 50)

Participants felt as if they had no choice but to suppress their emotions as a way of coping:

‘I became angry, but I didn’t show it to the family…’; ‘… you know you have to take your feelings aside …’ and ‘… keep it inside of me; you just let it go.’ (Participant 3, female, 27)

The suppressed emotions stay unresolved:

‘… what will you do about it? You do nothing about it…’ and can escalate into strong feelings: ‘What will you do? You will just hate …’ (Participant 4, female, 50).

Suppressing emotions have far-reaching consequences:

‘… so keeping it to myself it will cause me a lot of harm; for me and my family and those who know me, my friends …’ (Participant 8, female, 46).

Participants felt a sense of duty to suppress their personal emotions and provide help. They mentioned that the nurse was expected to always be in control:

‘This is what I have to do, that’s it …’; ‘… you have to pull yourself together … regain your strength to do your work.’ (Participant 4, female, 50)

‘I didn’t show (my feelings) to the family and relatives because I must comfort the patient. I must not be emotional in front of the patient. I must give hope to the patient, be supportive, though it was hurting to me. But I tried to be strong for the sake of the patient …’ (Participant 3, female, 27)

‘I have to carry on even though it is difficult for me … it’s my responsibility … I should provide help first for there’s no way that I cannot help a patient because of my emotions … I must just help out no matter how bad it can be …’ (Participant 4, female, 50).

‘… we have to deal with it (IPV) whether we like it or not, and we have to get some positive results from that …’ (Participant 4, female, 50).

### Accommodation

Participants used different ways to adapt to or accommodate the inevitable exposure to survivors of IPV in the emergency unit, namely, distraction, spirituality, acceptance and meaning making.

Distraction: Some participants sought distraction in the form of time-out after being emotionally upset by a specific case, for example:

‘… you just want to cool down a little bit … just need a little time …’; ‘… just go to the kitchen and grab something …’; ‘… listen to the music – whatever you can do to just relieve yourself’, and ‘… usually what I do I go outside. There are porters, so I just sit with them and they always make a noise, and I listen to whatever they are saying, and talk to them and come back to my work.’ (Participant 1, female, 32)

Small talk with co-workers also provided distraction:

‘… we are chatting about something different; they (the images of the injured survivor) can’t come back …’ (Participant 1, female, 32).

At home, participants’ family members provided distraction, for example:

‘… when I am playing with my daughters I am trying to forget what happened…’ (Participant 8, female, 46).

Spirituality: Participants used internal spiritual resources to help them cope:

‘… the first thing that I do before I can go to the patient, I am just taking a two minutes break to ask God for strength, and for power and suddenly … I have started to stand for that woman … and I think God also sees that my child is in trouble, let me give her the strength to do what she is suppose to do …’ (Participant 8, female, 46).

This participant found spiritual meaning in providing care to survivors of IPV:

‘… for us to be here is because of the purpose from God. He knows that there will be some other people outside who need our help so He will give us the strength almost every day so that we can help those people because they are not able to help themselves.’ (Participant 8, female, 46)

Acceptance: Although acceptance was described as a difficult process, some participants managed to come to terms with their experiences. For one participant, the death of a woman exposed to IPV was a particularly difficult situation to cope with; the description indicates how spirituality helped her to accept the inevitable:

‘… at the end I just told myself let me accept what has happened … but it’s difficult to accept it … I just told myself I have got a family I have to look after. I have got a job and I have patients that want my help … what is left is just to pray to God to let it not happen again. Sometimes it’s very hard to accept – you are in denial about what happened – but in the end you have to accept. It’s done, she’s gone; she’s gone and she can’t come back. I can’t walk around with it forever.’ (Participant 8, female, 46)

Meaning making: Participants aimed to answer three questions as they struggled to make meaning of IPV, namely:

‘Who is that person who can do these horrible things?’; ‘Why is the person still in this relationship?’ and ‘Why is it happening to us women, what went wrong?’ (Participant 2, female, 29)

A question that bothered participants concerned the reasons for a person hurting his intimate partner:

‘How can another person cause this pain … what drove them to inflict this pain on this other person?’ (Participant 2, female, 29)

Participants reflected on the contradictory behaviour perpetrators of IPV often display:

‘At the same time they tell the world that they love this other person and then this is definitely not the way to show that they love this person. Mm … It’s just so contradicting …’ (Participant 7, female, 26).

Some participants reasoned that a family history of violence can contribute to the perpetrator’s behaviour:

‘Sometimes the person was raised in a home where there was constant physical abuse and to them it’s the norm; it’s the way how you deal with anger … to turn to violence for solving a problem.’ (Participant 7, female, 26)

Emergency nurses provide care to seriously injured survivors knowing that:

‘the very same women who are abused in those relationships, they still stay … going to go back to that person …’ (Participant 1, female, 32).

Participants presented rationales for women to stay in relationships where they are exposed to violence:

‘… if I do this (leave) then I lose the man that I love …’ (Participant 9, female, 39)

‘… sometimes I told myself it’s because she’s not working, the reason why she keeps on staying in that relationship …’ (Participant 6, female, 36)

‘… this man was in serious power because of the occupation he was doing and the poor woman had nothing … he was so wild …’ (Participant 4, female, 50)

‘… men they have got this belief – especially in our culture – they will tell you: these things a woman is not supposed to do, and then they take advantage of this thing because they have got powers.’ (Participant 4, female, 50)

‘… she’s afraid to leave that relationship and go back home. At home there’s nobody working and she’ll go home with those kids and it’s going to be a burden on her parents …’ (Participant 6, female, 36)

‘… some of the families, especially we blacks, we have this tendency of not accepting other situations when somebody has failed (divorce) …’ (Participant 5, female, 35)

Gender inequalities forms part of the MDG 3 and participants linked cultural sanctioning and gender inequalities to IPV. Participants reflected on IPV as a social problem:

‘… you realise there’s been lots of people coming through, lots of organisations … but men are still abusive. And you ask yourself: these campaigns have existed for years but men still continue to abuse women in different ways.’ (Participant 2, female, 29)

## Discussion

It can be deduced from the findings that emergency nurses attempt to cope with the exposure to survivors of IPV, either through aiming to escape, or dealing with their experiences so as to fulfil their professional responsibilities.

Researchers propose many ways to conceptualise coping.^39^ The one that seemed to be most appropriate for this discussion concerns disengagement and engagement coping. When using disengagement coping, a person aims to escape feelings of distress^39^ through distancing oneself from the stressor and related thoughts and emotions.^40^ Disengagement coping is related to distress and poor mental health.^41^ Participants used escape as evidenced in behaviour to avoid caring for survivors of IPV, and a cognitive decision to detach themselves emotionally from survivors’ experiences. Ignoring or detaching oneself from a situation, a common way of coping used by nurses,^42^ is associated with depression in nurses,^11^ burnout in critical care nurses^44^ and an increase in somatic complaints in emergency nurses.^23^ Nurses who struggle to cope may find it difficult to empower survivors of IPV.

Engagement coping, a direct approach toward a stressor or related thoughts and emotions points to better adjustment and lower levels of distress than disengagement coping.^40,41^ Engagement coping can be further distinguished as primary or secondary control strategies. When using primary control strategies, a person attempts to change a negative situation through problem solving. Primary control also includes emotion-focused coping such as emotional expression, emotional regulation and support-seeking. Secondary control coping, or accommodative coping, involves strategies to adapt to current conditions such as distraction, acceptance and meaning-focused coping.^39,40^ Participants sought support from colleagues and spiritual counsellors. Nurses working in different settings use this form of informal debriefing or peer support.^42^ Peer and social support have a protective effect on the occurrence of post-traumatic stress symptoms in emergency nurses.^23,43^ Emergency nurses with secondary traumatisation who scored their relationships with colleagues as poor, or average, experienced interpersonal conflicts at work and were less likely to obtain social support.^31^

The different ways people use to regulate emotions may have different outcomes.^45^ In this research, participants attempted to regulate their emotional responses through suppression. Suppression, hiding what one is feeling or inhibiting the emotional experience,^46^ is regarded as a more dysfunctional way of emotion regulation. People vulnerable to depression, for example, tend to use suppression.^47^ The incongruence between what is experienced internally, and showed externally, has a negative effect on relationships.^45^ The nurses in this research indicated that they view suppression of emotions as a professional expectation. Nurses may moderate the connection with, and distance from, patients to help them regulate emotions and maintain professionalism. What appears as suppression may indeed be an attempt of nurses to manipulate emotional boundaries.^48^

Accommodation refers to the adjustments a person makes to cope with events that cannot be controlled.^39^ In the study, participants used accommodative coping by seeking distraction and using spirituality, acceptance and meaning making. The last three ways of coping are inter-related and resemble a process of cognitive restructuring or reappraisal.^13^ For example, accepting the death of a woman exposed to IPV was facilitated in the study through spirituality. Acceptance may also be considered as coping through meaning making as the person comes to terms with a stressful event.^49^ Positive cognitive restructuring is illustrated in finding strength in the idea that the work with survivors is a fulfilment of a spiritual purpose. Spirituality, also referred to as ‘religious coping’, helps people to find the strength to endure, stay calm and find meaning in stressful situations.^50^ Spirituality helps to avoid emotional burnout^51^ and is associated with resilience in nurses.^8^

Meaning making attempts to cognitively restructure, make sense of, or find benefit in adverse events.^49^ Human beings strive towards constructing situational and global meaning. Situational meaning refers to the way in which an event is appraised in terms of the relevance and implications it holds for a person. Global meaning is described in terms of beliefs, goals and subjective meaning.^49^ Stress arises when a discrepancy occurs between situational and global meanings.^52,55^ Traumatic experiences disrupt global meanings. To make sense of traumatic experiences and alleviate the distress a person may use cognitive processing to alter the meaning attached to the event or alter his or her global beliefs to accommodate the event.^49^

Trying to find meaning in the senselessness and suffering associated with IPV, participants either changed their global beliefs or the situational meanings they attached to IPV in order to avoid emotional distress. Participants tried to find an explanation behind the behaviour of the perpetrator and the woman who stays in the violent relationship. They ascribed the perpetrator’s violence to a violent upbringing and the women’s reluctance to leave as caring for her children or family. They reappraised their understanding of intimate relationships within the context of culture, socio-economic factors and gender inequalities. Ongoing discrepancies between global and appraised meaning may alter a person’s belief system, a characteristic of vicarious traumatisation.^52^

## Recommendations

The dimensions of knowledge development in nursing may provide a way for emergency nurses to challenge their current ways of coping and take corrective action.^53^ It is recommended that emergency nurses use the personal knowing processes^53^ to critique and become more mindful of their current ways of coping with the exposure to survivors of IPV. The personal knowing processes focus on integrating mind, body and spirit to create inner calm and on facilitating congruence between intentions and actions. Critiquing analyses the status quo, building on nurses’ ability to recognize a disadvantaged position and imagine ways in which the current situation can be improved.^53^

Reflection may help to develop health care professionals’ coping skills, protect nurses against burnout and enhance emotional resilience.^54,55^ Emergency nurses may use reflection to bring current ways of coping into conscious awareness, become mindful of the consequences and effectiveness of these, share and strengthen constructive ways of coping, critique less effective ways of coping and put together their experience and resources to explore, generate and develop alternative ways of coping with the exposure to survivors of IPV.^53^ The outcome is visualised as the integration of ways of coping that will relieve emotional distress; for example, engagement coping, seeking support and emotion regulation.

Nurses vulnerable to secondary traumatisation may benefit from prevention programmes that facilitate effective, problem-focused coping, emotion regulation, constructive reappraisal^56^ and meaning making practices.^57^ Understanding meaning making may help nurses to avoid internalising negative meaning that may result in distress.^58^ A formal system of peer support in emergency units is recommended,^18^ essentially to provide empathy and listening and to refer nurses who may be at risk for secondary traumatisation to professional help.^59^

## Conclusion

Emergency nurses may play an important role in attaining MDG 3 by identifying and empowering women exposed to IPV. Emergency nurses used a variety of ways to cope with the exposure to survivors of IPV. Engagement coping seems to be more constructive than disengagement coping, for example, seeking help versus avoidance. There seems to be a thin line between emotional suppression, regarded as dysfunctional coping, and manipulation of emotional boundaries to maintain professionalism. Accommodative coping may be functional; for example, acceptance facilitated by spirituality, whilst attempts at meaning making that disrupt global beliefs can give way to vicarious traumatisation. Coping in the study context was individual and ‘silent’ as emergency nurses tend to not discuss their ways of coping. The lack of formal structures to help emergency nurses cope with the exposure to survivors of IPV put nurses at risk for vicarious traumatisation. Impeding nurses’ ability to empower survivors of IPV, vicarious traumatisation may be a potential stumbling block in reaching MDG 3: promoting gender equality and empowering women.
